# Sex-specific differences in the association of dietary riboflavin intake with depression and suicidal ideation

**DOI:** 10.3389/fnut.2025.1587322

**Published:** 2025-09-09

**Authors:** Haorui Chu, Dongyu Liu, Wei Peng, Tianci Qiao

**Affiliations:** ^1^The First Clinical School, Shandong University of Traditional Chinese Medicine, Jinan, China; ^2^Department of Neurology, Affiliated Hospital of Shandong University of Traditional Chinese Medicine, Jinan, China; ^3^Department of Geriatric Medicine, Affiliated Hospital of Shandong University of Traditional Chinese Medicine, Jinan, China

**Keywords:** sex-specific, dietary riboflavin, depression, suicidal ideation, cross-sectional study

## Abstract

**Background:**

Attention is increasingly focused on the relationship between dietary micronutrients and affective disorders. Currently, the association of riboflavin intake with depression and suicidal ideation is controversial. Therefore, the present study utilized a large population-based database to explore these relationships.

**Methods:**

We included 29,466 participants. The study data was weighted for analysis. The associations of dietary riboflavin intake with depression and suicidal ideation were examined using logistic regression models. To elucidate the dose-response relationship of dietary riboflavin intake with outcomes, we used restricted cubic splines. Recursive methods were utilized to identify the inflection point. Subsequently, stratified analyses were conducted to investigate differences across subgroups.

**Results:**

After adjusting for all confounding factors, the ORs (95% CIs) for the association of dietary riboflavin intake with depression and suicidal ideation were 1.02 (0.93, 1.11) and 1.09 (0.93, 1.28), with no statistical significance. The restricted cubic splines indicated non-linear associations, and the relationship of dietary riboflavin intake with depression was inverse only before the inflection point (1.44 mg/day). In the relationship with suicidal ideation, significant association was found both before and after the inflection point (1.42 mg/day). Furthermore, sex-specific subgroup differences were found.

**Conclusions:**

In this large-sample study, the non-linear association of dietary riboflavin intake with depression and suicidal ideation was found. Furthermore, differences in this relationship based on sex were observed.

## Introduction

Depression is a widespread public health concern that exerts a substantial impact on individuals and societies worldwide (1). The World Health Organization estimates that over 300 million people globally are impacted by depression, which ranks it as one of the most significant health challenges globally (2, 3). The implications of depression extend far beyond the realm of mental health; it is closely associated with various long-term physical health problems, such as cardiovascular diseases, chronic respiratory conditions, and diabetes (4). Moreover, individuals with depression face a significantly elevated risk of suicide, highlighting its severe implications for both personal health and societal wellbeing (5). Therefore, understanding and effectively treating depression is crucial for enhancing global public health.

Traditionally, research on depression has focused on psychosocial factors and neurobiological mechanisms. Although antidepressants are the primary mode of treatment for depression, they are often accompanied by adverse effects and demonstrate limited efficacy across diverse patient populations (6). These limitations have prompted growing interest in alternative or complementary therapeutic strategies, particularly dietary interventions. In recent years, the relationship between nutrients and depression has increasingly gained attention. Inadequate dietary nutrition is considered a potential risk factor for depression, especially in cases of insufficient micronutrient intake (7, 8). A study has demonstrated an association between low vitamin C intake and an increased risk of depression (9). A meta-analysis revealed that increased dietary intake of vitamin E was negatively related to depression (10). Therefore, further research into the association between dietary micronutrients and depression is warranted to support the development of targeted dietary strategies for managing depression and reducing suicide risk.

Riboflavin, also known as vitamin B2, is an essential member of the B-vitamin family and plays multiple key roles in human health (11, 12). It serves as a crucial coenzyme in cellular metabolic processes, particularly in energy metabolism and red blood cell formation (13). Additionally, riboflavin is vital for the health of the nervous system. Studies suggest that it may influence the synthesis of neurotransmitters, such as serotonin and norepinephrine, which are closely linked to emotional regulation and cognitive function (14). Findings from the Global Dietary Database showed that more than 4 billion people were not intaking enough riboflavin through their diets (15). There is evidence to suggest that a deficiency in riboflavin may be associated with the onset of depression (16). However, a prospective study from Iran revealed riboflavin was not associated with depression (17). A separate study found sex differences in the relationship (18). Given these inconsistencies, the relationship between dietary riboflavin intake and depression remains unclear. Therefore, the present study aimed to explore the association of dietary riboflavin intake with depression and suicidal ideation utilizing data from the National Health and Nutrition Examination Survey (NHANES).

## Methods

### Study population

**Figure 1:**
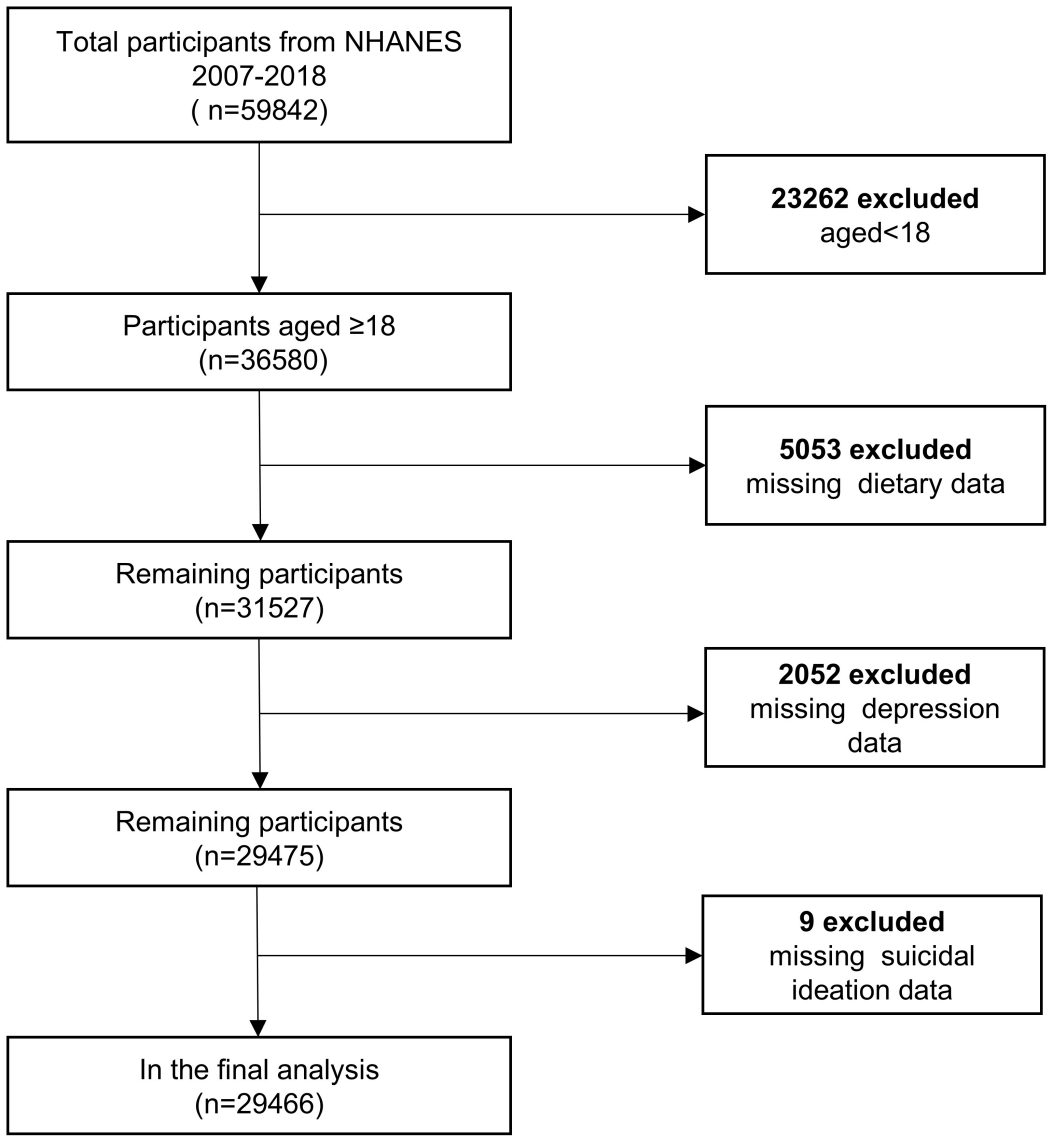
Flow chart of sample selection.

The NHANES is an ongoing health and nutrition survey conducted by the National Center for Health Statistics (NCHS). Its methodology combines interviews, physical examinations, and laboratory tests. The interviews typically encompass queries about health status, health behaviors, and dietary habits. Physical examinations and laboratory tests are conducted in a specialized mobile examination center (MEC). Participants must fast for at least 9 h. All anthropometric indexes were measured at the MEC by standardized protocols (19). Laboratory tests included the collection of blood and urine samples. Data are collected annually and released periodically, representing the general U.S. population. This aids government and research institutions in monitoring changes in public health and nutrition. The NHANES data are accessible to researchers and the public. The survey protocol has been approved by the NCHS Research Ethics Review Board (ERB). Informed consent was provided by all participants. Our analysis included 29,466 participants after exclusions based on the following criteria: (1) age under 18 years old; (2) missing data on riboflavin intake; (3) missing data on depression; (4) missing data on suicidal ideation (Figure 1).

### Assessment of riboflavin intake

The NHANES utilizes a comprehensive approach to collect dietary information, employing both a 24-h dietary recall method and the Food Frequency Questionnaire (FFQ) (20). This methodology entails conducting two separate 24-h dietary recall assessments for each participant. The initial dietary recall is executed through a face-to-face interview, followed by a secondary recall via telephone within a 3-to 10-day interval. The aggregation of daily nutritional intake, encompassing caloric content and other essential dietary components from all consumed food and beverages, is accurately calculated through the sophisticated United States Department of Agriculture (USDA) database, specifically designed for dietary research (21, 22). The intake of dietary riboflavin is meticulously determined by averaging the data obtained from these two dietary recalls.

### Assessment of depression and suicidal ideation

Depression was assessed using the Patient Health Questionnaire-9 (PHQ-9), a survey instrument developed in accordance with the Diagnostic and Statistical Manual of Mental Disorders, Fourth Edition (DSM-IV). Its reliability in both cross-sectional and clinical studies is widely recognized and well-established. Participants were asked how often they had experienced depressive symptoms in the past 2 weeks. A scale ranging from 0 to 3 was used for each item, yielding a total score range of 0–27. In this study, a PHQ-9 score of ≥10 was employed as the threshold for identifying depression, offering a sensitivity and specificity of 88% (23). Suicidal ideation was assessed through the questions on suicidal thoughts in the PHQ-9 (24, 25).

### Covariates

To exclude the influence of potential confounders on the study's findings, a comprehensive set of covariates was meticulously adjusted. Demographic characteristics ascertained via household interviews included age, sex, race, educational level, marital status, and income-to-poverty ratio. Lifestyle incorporated smoking status, drinking status, and vigorous recreational activities. Additionally, the inclusion of chronic diseases commonly associated with depression, such as hypertension, diabetes, cancer, stroke, and cardiovascular disease (CVD), was integral to our analysis. Body mass index (BMI) and waist were ascertained from physical measurements. C-reactive protein (CRP) and dietary micronutrient intake were also included for adjustment.

### Statistical analysis

Given the sampling survey methodology employed by NHANES, we conducted weighted analyses on the data to ensure the results were representative of the American population. For baseline characteristic analysis, the study population was divided into four groups based on quartiles of riboflavin intake. Statistical differences between groups were assessed using one-way analysis of variance (ANOVA) for continuous variables and chi-square test for categorical variables. For continuous variables, data was presented as means ± standard error (SE), and for categorical variables, as percentages (SE).

In this study, depression was the dichotomous variable. Logistic regression models were used to examine the association between riboflavin intake and outcomes. Additionally, to adequately eliminate the effect of confounding factors on the results, three logistic regression models were established: an unadjusted model, a model adjusted for age, sex, and race, and a further adjusted model including all additional potential confounders. Furthermore, restricted cubic splines were used to assess the potential for a non-linear relationship of dietary riboflavin intake with depression and suicidal ideation, allowing for flexibility in modeling the relationship and detecting threshold effects. Threshold levels of riboflavin intake were determined using a recursive method. The piecewise linear regression models were compared with the linear model using the log-likelihood ratio test. Furthermore, we performed a comprehensive subgroup analysis to explore whether subgroup differences existed. All analyses were performed using the statistical software R (version 4.2.1) and Empower Statistics software. A two-tailed *P-value* < 0.05 was considered statistically significant.

## Results

### Baseline characteristics

Table 1 presents the general characteristics of the study population segmented by quartiles of riboflavin intake. The average age of participants was 46.79 (0.25) years. The proportion of men and women was 47.85 and 52.15%, respectively. Compared to other groups, participants with the highest intake of riboflavin were more likely to be men, White, possess an education beyond high school and higher, income-to-poverty ratios, former alcohol consumers, and engage in vigorous recreational activities. They also tended to have a lower prevalence of chronic diseases and lower levels of BMI and CRP. Additionally, their intake of other dietary micronutrients was higher.

**Table 1:** Population characteristics according to riboflavin intake quartiles.

**Variable**	**Total (*n* = 29,466)**	**Q1 (*n* = 7,375)**	**Q2 (*n* = 7,367)**	**Q3 (*n* = 7,359)**	**Q4 (*n* = 7,365)**	***P*-value**
Age (years)	46.79 (0.25)	45.14 (0.34)	47.45 (0.36)	48.04 (0.32)	46.27 (0.31)	< 0.0001
**Sex (%)**						< 0.0001
Men	47.85 (0.01)	33.31 (0.69)	39.94 (0.77)	48.50 (0.65)	63.52 (0.73)	
Women	52.15 (0.01)	66.69 (0.69)	60.06 (0.77)	51.50 (0.65)	36.48 (0.73)	
**Race/ethnicity (%)**						< 0.0001
Mexican American	8.56 (0.01)	9.49 (0.89)	9.51 (0.88)	8.26 (0.76)	7.43 (0.66)	
White	67.00 (0.03)	52.45 (1.74)	64.12 (1.60)	71.07 (1.32)	75.61 (1.23)	
Black	11.22 (0.01)	20.28 (1.31)	11.89 (0.81)	8.76 (0.60)	6.69 (0.49)	
Other	13.22 (0.01)	17.77 (0.86)	14.47 (0.79)	11.91 (0.63)	10.28 (0.62)	
**Education level (%)**						< 0.0001
Less than high school	4.72 (0.00)	7.01 (0.41)	5.28 (0.40)	4.10 (0.27)	3.28 (0.25)	
High school	34.50 (0.01)	41.70 (1.14)	35.08 (0.98)	32.35 (0.86)	31.09 (1.09)	
More than high school	60.72 (0.02)	51.29 (1.17)	59.63 (1.13)	63.54 (0.93)	65.63 (1.18)	
**Marital status (%)**						< 0.0001
Married/living with partner	61.40 (0.02)	54.23 (0.93)	64.03 (0.92)	66.83 (0.77)	66.23 (0.89)	
Divorced/separated/widowed	17.79 (0.00)	22.27 (0.64)	19.09 (0.52)	17.91 (0.61)	15.66 (0.55)	
Never married	17.54 (0.01)	23.50 (0.87)	16.88 (0.80)	15.26 (0.68)	18.11 (0.74)	
**Smoking status (%)**						< 0.0001
Current	18.63 (0.01)	21.40 (0.77)	17.73 (0.70)	17.20 (0.70)	19.80 (0.68)	
Former	24.29 (0.01)	19.81 (0.69)	23.82 (0.78)	25.82 (0.72)	27.72 (0.75)	
Never	55.42 (0.01)	58.78 (0.97)	58.45 (0.86)	56.98 (0.94)	52.48 (0.84)	
**Drinking status (%)**						< 0.0001
Never	11.02 (0.00)	17.20 (0.73)	12.44 (0.52)	10.24 (0.62)	8.00 (0.52)	
Former	12.03 (0.00)	13.29 (0.56)	12.61 (0.66)	12.22 (0.56)	12.29 (0.57)	
Current	72.84 (0.02)	69.52 (0.99)	74.95 (0.90)	77.54 (0.86)	79.71 (0.84)	
**Vigorous recreational activities (%)**						< 0.0001
Yes	26.47 (0.01)	20.84 (0.79)	24.45 (0.88)	26.76 (0.92)	31.66 (1.14)	
No	73.53 (0.02)	79.16 (0.79)	75.55 (0.88)	73.24 (0.92)	68.34 (1.14)	
**Stroke (%)**						< 0.0001
Yes	2.88 (0.00)	3.84 (0.28)	3.52 (0.25)	2.97 (0.20)	1.97 (0.20)	
No	93.80 (0.02)	96.16 (0.28)	96.48 (0.25)	97.03 (0.20)	98.03 (0.20)	
**CVD (%)**						< 0.0001
Yes	8.56 (0.00)	9.85 (0.52)	10.05 (0.49)	9.04 (0.39)	7.04 (0.38)	
No	88.19 (0.02)	90.15 (0.52)	89.95 (0.49)	90.96 (0.39)	92.96 (0.38)	
**Hypertension (%)**						< 0.0001
Yes	37.50 (0.01)	38.90 (0.96)	39.26 (0.81)	37.95 (0.84)	34.73 (0.84)	
No	62.50 (0.01)	61.10 (0.96)	60.74 (0.81)	62.05 (0.84)	65.27 (0.84)	
**Diabetes (%)**						0.005
Yes	10.62 (0.00)	11.72 (0.58)	11.37 (0.45)	10.79 (0.53)	9.49 (0.43)	
No	88.29 (0.02)	88.28 (0.58)	88.63 (0.45)	89.21 (0.53)	90.51 (0.43)	
Yes	10.33 (0.00)	9.94 (0.56)	10.01 (0.55)	12.59 (0.51)	9.98 (0.55)	
No	86.36 (0.02)	90.06 (0.56)	89.99 (0.55)	87.41 (0.51)	90.02 (0.55)	
**Depression (%)**						< 0.0001
Yes	8.00 (0.00)	10.99 (0.51)	7.93 (0.45)	7.21 (0.35)	6.69 (0.41)	
No	92.00 (0.02)	89.01 (0.51)	92.07 (0.45)	92.79 (0.35)	93.31 (0.41)	
**Suicidal ideation (%)**						< 0.0001
Yes	3.28 (0.00)	4.44 (0.31)	3.35 (0.28)	2.44 (0.21)	3.19 (0.27)	
No	96.72 (0.02)	95.56 (0.31)	96.65 (0.28)	97.56 (0.21)	96.81 (0.27)	
CRP (mg/dl)	2.20 (0.07)	2.78 (0.17)	2.44 (0.12)	2.16 (0.12)	1.66 (0.08)	< 0.0001
BMI (kg/m^2^)	29.09 (0.09)	29.38 (0.14)	29.10 (0.13)	29.17 (0.13)	28.81 (0.11)	0.002
Waist (cm)	99.21 (0.24)	98.43 (0.34)	98.55 (0.35)	99.68 (0.32)	99.83 (0.31)	< 0.001
Income-to-poverty ratio	3.00 (0.04)	2.49 (0.04)	2.94 (0.05)	3.19 (0.04)	3.22 (0.05)	< 0.0001
Niacin intake (mg/day)	24.88 (0.12)	14.71 (0.15)	20.46 (0.16)	25.66 (0.17)	34.67 (0.24)	< 0.0001
Vitamin C intake (mg/day)	79.05 (1.09)	55.12 (1.31)	72.34 (1.48)	83.09 (1.54)	97.17 (1.84)	< 0.0001
Vitamin E intake (mg/day)	8.56 (0.08)	5.10 (0.08)	7.25 (0.09)	8.93 (0.10)	11.63 (0.13)	< 0.0001

Mean ± SE for continuous variables;% (SE) for categorical variables.

### Association between exposure and outcomes

**Figure 2:**
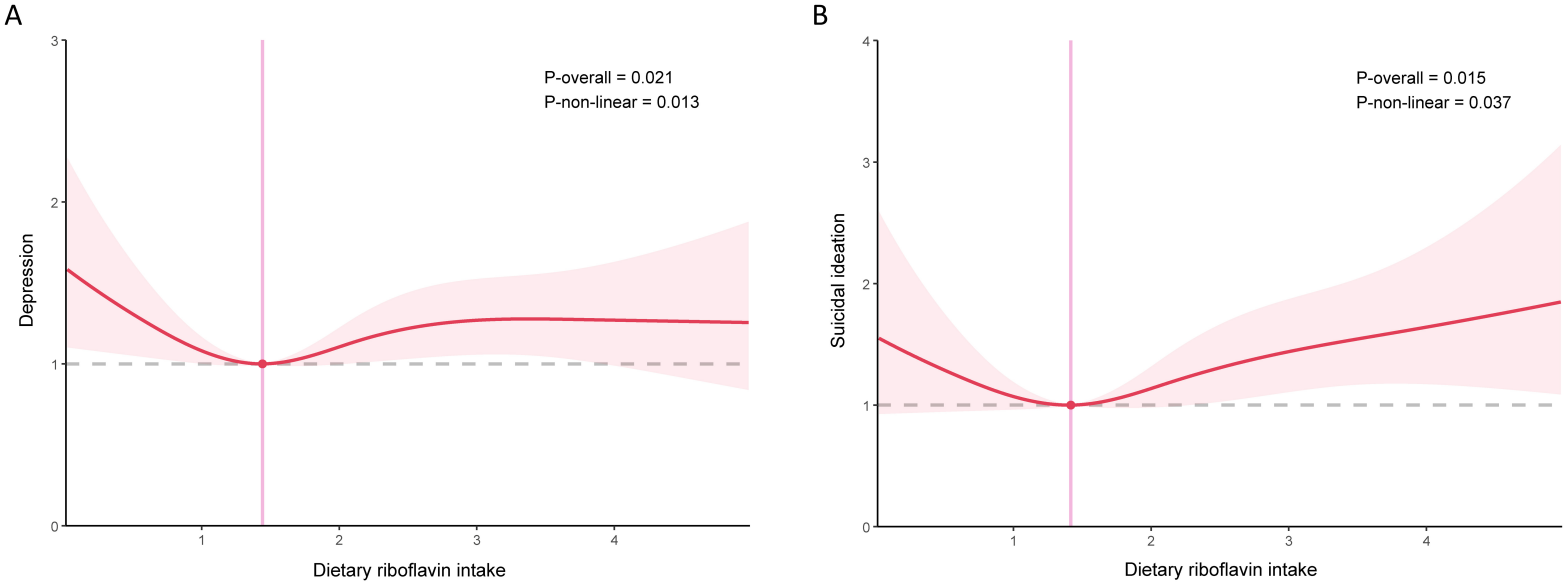
Association of dietary riboflavin intake with depression and suicidal ideation **(A)** depression; **(B)** suicidal ideation. Age, sex, race, education level, income-to-poverty ratio, marital status, smoking status, drinking status, vigorous recreational activity, BMI, waist, stroke, diabetes, CVD, cancer, hypertension, vitamin C intake, niacin intake, vitamin E intake, and CRP were adjusted.

To control the influence of confounding factors, three logistic regression models were constructed (Table 2). Model 1 made no adjustments for covariates; the basic demographic factors were adjusted for in Model 2; and all covariates were adjusted for in Model 3. In the model with fine adjustments for covariates (Model 2), dietary riboflavin intake was negatively correlated with depression and suicidal ideation. However, after adjustment for all confounders (Model 3), the ORs (95% CIs) were 1.02 (0.93, 1.11) and 1.09 (0.93, 1.28), indicating no statistical significance (Table 2). In the further sensitivity analyses, using Q3 as the reference, ORs (95% CIs) were >1 in all other groups, suggesting the potential non-linear associations between the exposure and outcomes. Furthermore, we employed restricted cubic splines to test for the dose-response associations, confirming the non-linear patterns (Figure 2). The inflection point was calculated to be 1.44 mg/day in the association between dietary riboflavin intake and depression. Before this point, riboflavin intake was negatively correlated with depression [0.73 (0.57, 0.93)] (Table 3). Beyond this inflection point, the positive correlation showed no statistical significance with ORs (95% CIs) of 1.09 (0.98, 1.20; Table 3). In the association between dietary riboflavin intake and suicidal ideation, significant associations were found both before the inflection point [0.60 (0.36, 1.00)] and after the inflection point [1.17 (1.03, 1.33)].

**Table 2:** Association of dietary riboflavin intake with depression and suicidal ideation.

	**Model 1 OR (95% CI)**	**Model 2 OR (95% CI)**	**Model 3 OR (95% CI)**
**Depression**			
Dietary riboflavin intake	0.82 (0.77, 0.88)	0.88 (0.83, 0.94)	1.02 (0.93, 1.11)
Q3	1.0	1.0	1.0
Q1	1.59 (1.38, 1.83)	1.44 (1.26, 1.66)	1.19 (0.96, 1.48)
Q2	1.11 (0.95, 1.30)	1.06 (0.90, 1.24)	1.10 (0.88, 1.37)
Q4	0.92 (0.81, 1.06)	1.00 (0.87, 1.15)	1.25 (0.99, 1.57)
*P* for trend	< 0.0001	< 0.0001	0.63
**Sex**			
Men	0.86 (0.78, 0.95)	0.87 (0.79, 0.96)	0.98 (0.83, 1.15)
Women	0.89 (0.82, 0.96)	0.90 (0.83, 0.97)	1.05 (0.93, 1.18)
**Suicidal ideation**			
Dietary riboflavin intake	0.88 (0.80, 0.97)	0.90 (0.82, 0.99)	1.09 (0.93, 1.28)
Q3	1.0	1.0	1.0
Q1	1.86 (1.49, 2.33)	1.81 (1.44, 2.28)	1.50 (0.96, 2.34)
Q2	1.39 (1.08, 1.78)	1.37 (1.07, 1.76)	1.32 (0.88, 1.97)
Q4	1.32 (1.06, 1.64)	1.34 (1.07, 1.67)	1.87 (1.36, 2.58)
*P* for trend	0.004	0.02	0.21
**Sex**			
Men	0.92 (0.80, 1.04)	0.93 (0.82, 1.05)	1.10 (0.91, 1.32)
Women	0.86 (0.73, 1.01)	0.87 (0.73, 1.02)	1.10 (0.83, 1.44)

Model 1: no covariates were adjusted.

Model 2:.age, sex, race were adjusted.

Model 3:age, sex, race, education level, income-to-poverty ratio, marital status, smoking status, drinking status, vigorous recreational activity, BMI, waist, stroke, diabetes, CVD, cancer, hypertension, vitamin C intake, niacin intake, vitamin E intake, and CRP were adjusted.

In the subgroup analysis stratified by sex, the model is not adjusted for the stratification variable itself.

**Table 3:** Threshold effect analysis of dietary riboflavin intake on depression and suicidal ideation using a two-piecewise linear regression model.

	**Adjust OR (95% CI)**	***P*-value**
**Depression**		
Fitting by standard linear model	1.02 (0.93, 1.11)	0.72
Fitting by two-piecewise linear model		
Inflection point	1.44	
< 1.44	0.73 (0.57, 0.93)	0.01
>1.44	1.09 (0.98, 1.20)	0.09
Log-likelihood ratio	0.005	
**Suicidal ideation**		
Fitting by standard linear model	1.09 (0.93, 1.28)	0.27
Fitting by two-piecewise linear model		
Inflection point	1.42	
< 1.42	0.60 (0.36, 1.00)	0.04
>1.42	1.17 (1.03, 1.33)	0.01
Log-likelihood ratio	0.019	

Age, sex, race, education level, income-to-poverty ratio, marital status, smoking status, drinking status, vigorous recreational activity, BMI, waist, stroke, diabetes, CVD, cancer, hypertension, vitamin C intake, niacin intake, vitamin E intake, and CRP were adjusted.

**Figure 3:**
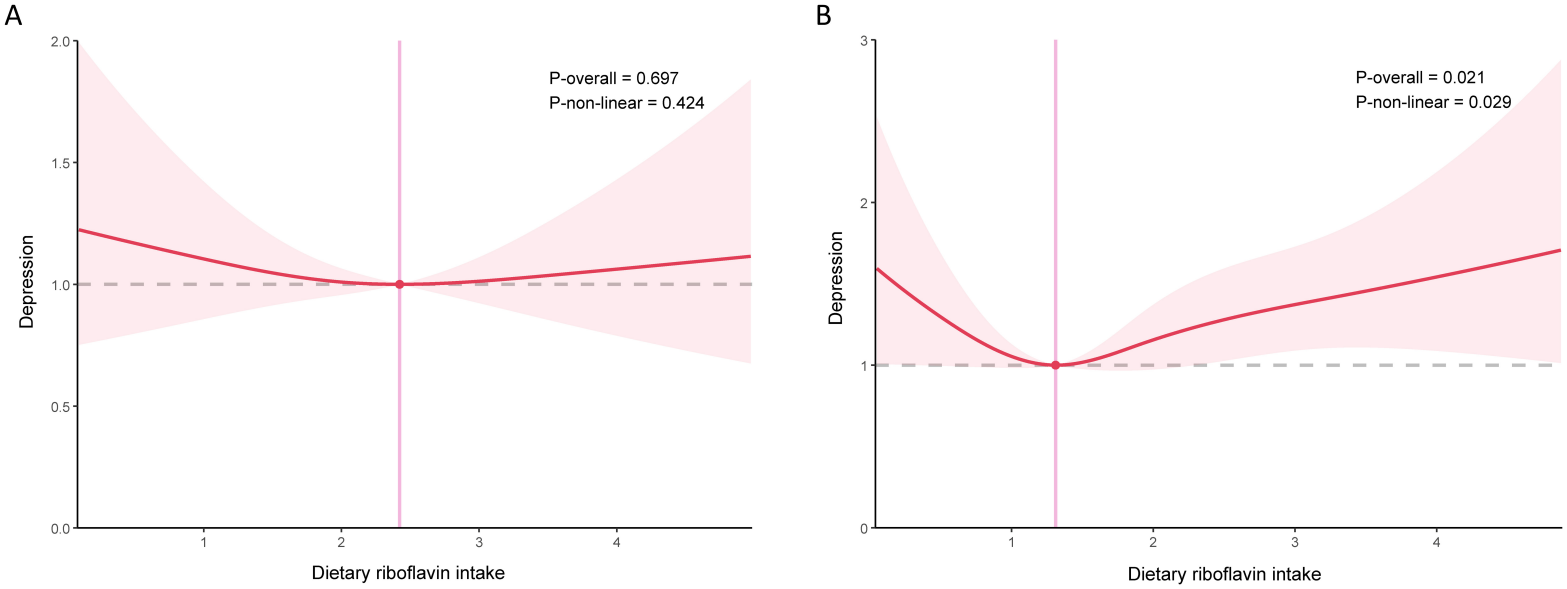
Association between dietary riboflavin intake and depression stratified by sex **(A)** men; **(B)** women.

**Figure 4:**
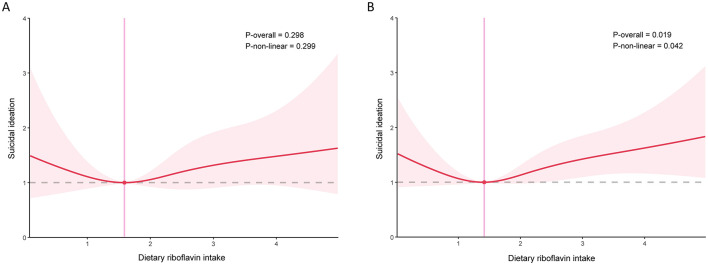
Association between dietary riboflavin intake and suicidal ideation stratified by sex **(A)** men; **(B)** women.

**Figure 5:**
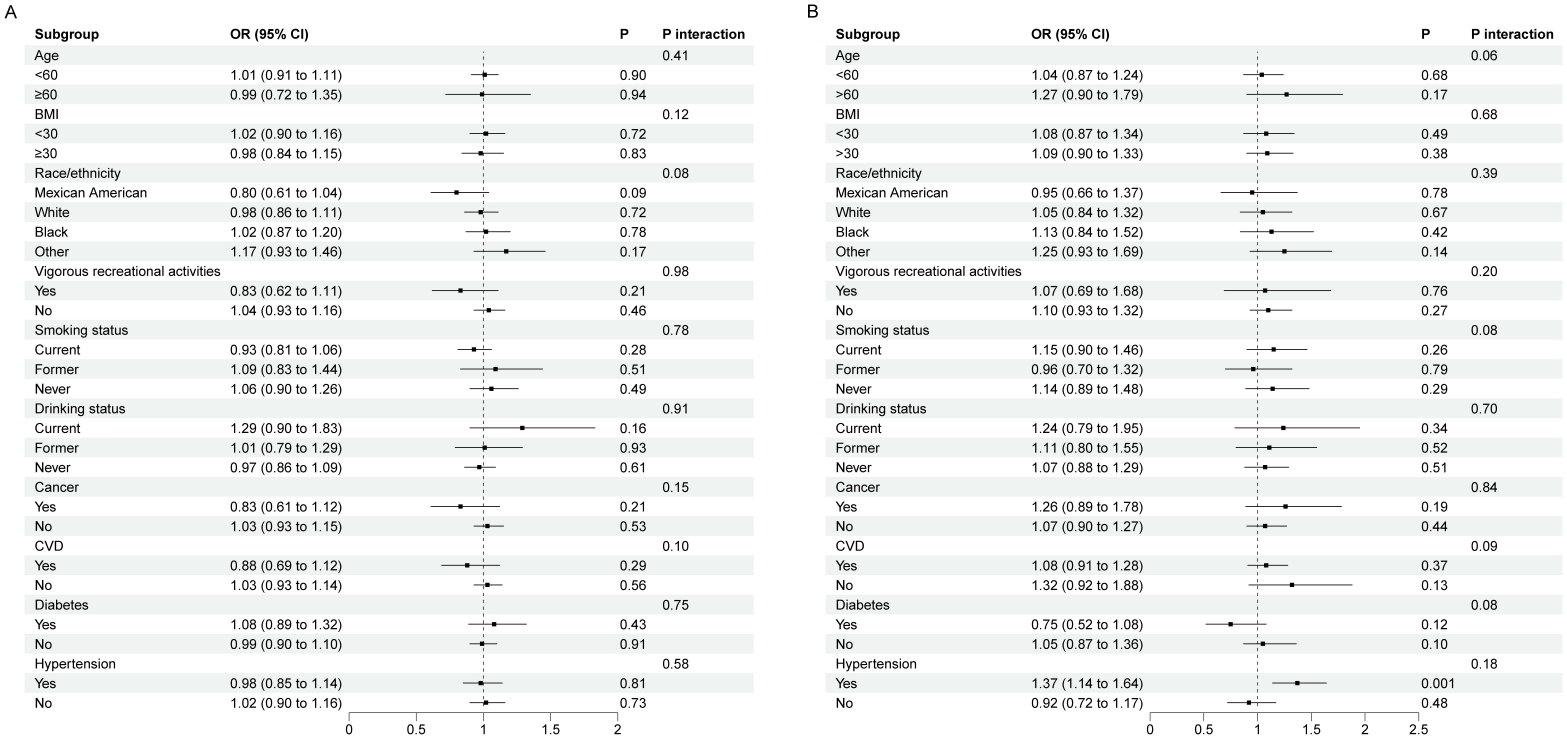
Subgroup analysis of association between dietary riboflavin intake with depression and suicidal ideation **(A)** depression; **(B)** suicidal ideation.

To further investigate whether there were subgroup differences in the association of dietary riboflavin intake with depression and suicidal ideation, stratified analyses were performed. In models adjusted for all covariates, the association of dietary riboflavin intake with depression was negative in men and positive in women, but neither was statistically significant (Table 2). After performing restricted cubic splines, the association between riboflavin intake and depression in women was found to be non-linear (Figure 3). In the association between dietary riboflavin intake and suicidal ideation, again no significant relationship was found (Table 2). Further restricted cubic splines revealed a *U*-shaped association between riboflavin intake and suicidal ideation among women, consistent with the total population (Figure 4). No significant interactions were observed in the further subgroup analyses (Figure 5).

## Discussion

In this cross-sectional study, we investigated the association of dietary riboflavin intake with depression and suicidal ideation among the adult population in the United States. After accounting for all included confounding factors, riboflavin intake was not significantly associated with depression and suicidal ideation. To explore the possibility of a non-linear relationship influencing the overall results, the restricted cubic spline was conducted, which revealed the non-linear associations of dietary riboflavin intake with depression and suicidal ideation in the general population. Stratified restricted cubic splines indicated that the associations were non-linear in women. To the best of our knowledge, this study represents the largest sample size to explore the relationship of dietary riboflavin intake with depression and suicidal ideation in the American population.

Previous research examining the relationship of dietary riboflavin intake with depression and suicidal ideation has yielded mixed results. A study of the Chinese elderly population revealed that dietary riboflavin intake was negatively correlated with depression (26). Another research from adults also supported the finding of a negative correlation (27). However, a study conducted on Japanese adults found no clear association of riboflavin intake with depression (28), aligning with our findings prior to the application of restricted cubic splines. Similarly, studies from Finland, Iran, and Australia also concluded that riboflavin intake was not associated with the onset of depression (17, 29, 30). Notably, a meta-analysis has indicated that riboflavin intake is significantly associated with depression only in women (31). Likewise, a Japanese study indicated that moderate intakes of riboflavin could prevent postpartum depression (32). In line with this, the Ryukyus Child Health Study suggested that the negative correlation between riboflavin intake and depressive symptoms was only present in girls (18). Conversely, a study on Korean adolescent girls found no association between riboflavin intake and depressive symptoms (33). An American study identified a *U*-shaped relationship between riboflavin intake and depression (34). However, this study had a relatively small sample size and limited covariates included, and it did not explore the effects before and after the inflection point or subgroup differences. A study with Korean subjects found an *L*-shaped relationship between dietary riboflavin intake and suicidal ideation and showed a consistent relationship in the sex subgroup, which was significantly different from our findings of sex differences (35).

The precise biological mechanisms underlying the relationship of specific levels of riboflavin intake with reduced risk of depression and suicidal ideation remain elusive, but they may be related to several potential mechanisms. First is the regulation of neurotransmitter synthesis. Riboflavin serves as a co-factor for various enzymes involved in the synthesis of numerous neurotransmitters, such as serotonin and dopamine (36). These neurotransmitters play a significant role in emotional regulation and the pathology of depression (37). An adequate supply of riboflavin can ensure the effective synthesis of these neurotransmitters, potentially exerting a positive effect in mitigating depression and suicidal ideation. Second is the reduction of oxidative stress. Riboflavin plays an important role in the cellular antioxidative defense mechanisms (38). Oxidative stress is believed to be linked to the pathogenesis of depression. By diminishing oxidative stress, riboflavin may aid in alleviating neuroinflammation and cellular damage associated with depression. Third is the improvement of energy metabolism. Riboflavin is a critical component of the mitochondrial electron transport chain, essential for maintaining cellular energy metabolism (39). Mitochondrial dysfunction is common in individuals with depression and suicidal ideation. Through enhancing mitochondrial function, riboflavin might help boost energy metabolism in brain cells, thereby ameliorating symptoms of depression. Fourth is the modulation of inflammatory responses. Recent research has established a link between depression and chronic low-grade inflammation (40). Riboflavin may play a role in modulating immune system responses by reducing the production of pro-inflammatory cytokines, thereby exerting an anti-inflammatory effect (41). Specifically, riboflavin is thought to affect the nuclear factor-kappa B (NF-κB) pathway, a crucial pro-inflammatory signaling cascade (42). By inhibiting NF-κB activation, riboflavin might potentially reduce the release of pro-inflammatory cytokines, thus mitigating inflammatory responses and alleviating depressive symptoms. Fifth is the neuroprotective action. Riboflavin is believed to offer neuroprotection through several mechanisms. It may reduce neuronal damage and apoptosis while promoting neuronal growth and regeneration. Neuronal injury and cell death are key elements in the pathology of depression (43). These mechanisms collectively highlight the potential multifaceted role of riboflavin in managing depression and suicidal ideation. Although further empirical studies are required to validate these effects, the outlined theoretical frameworks significantly contribute to our understanding of the relationship of dietary riboflavin intake with depression and suicidal ideation.

In our stratified analysis, we observed that only among women did riboflavin intake exhibit a non-linear relationship with depression and suicidal ideation. The hormonal composition in women, particularly estrogen, may influence the metabolism of riboflavin and its subsequent effects on the brain (44). Estrogen could enhance the role of riboflavin in neurotransmitter synthesis, thereby affecting emotional regulation and reducing the risk of depression and suicide in women. Oxidative stress and inflammatory responses are widely recognized as being associated with the onset of depression and suicidal ideation (45). Fluctuating hormone levels in women typically exacerbate oxidative stress and inflammatory responses, thereby increasing the risk of depression (46). The antioxidant and anti-inflammatory effects of riboflavin may protect against depressive symptoms in women by modulating these hormone-related oxidative stress and inflammatory responses. In addition, women are more likely to show emotional responses and psychological vulnerability in the face of stress, whereas riboflavin may regulate emotional states by influencing the synthesis and metabolism of neurotransmitters (e.g., serotonin, dopamine), which in turn affects suicidal ideation.

This study possesses several notable strengths. First, the NHANES database covers a broadly representative sample of the U.S. population. Such extensive coverage enhances the generalizability of the findings and provides a basis for investigating depression-related risk factors in different populations. Second, NHANES has a rigorous and standardized data collection protocol, which minimizes measurement error and ensures the accuracy and reliability of the collected data. Third, NHANES encompasses multidimensional information on diet, lifestyle, physiological indicators, socioeconomic status, and chronic diseases. Various potential confounders can be included to control the influence of covariates on the study results. Fourth, using the largest available sample, this study identified a non-linear association of dietary riboflavin intake with depression and suicidal ideation. Furthermore, sex-specific differences were observed. These findings provide a basis for identifying high-risk populations and offer important implications for public health policy. They may inform the development of dietary guidelines, guide targeted interventions for vulnerable groups, support the implementation of preventive strategies, and promote ongoing surveillance and future research.

Several limitations of this study should be acknowledged. First, due to the cross-sectional nature of the research, we can only observe associations between variables and cannot confirm whether one factor causes a change in another. Thus, it is difficult to determine causality. Future longitudinal studies are needed to elucidate causal relationships. Second, the data in NHANES, particularly about diet and lifestyle, relies on self-reported information from participants, which may be subject to memory biases or reporting inaccuracies. Third, as this study is based on the adult population in the United States, whether its conclusions are applicable to other countries requires further exploration. Finally, although this study has comprehensively included a range of covariates to mitigate the effects of confounders, due to limitations in the database, the impact of unaccounted covariates on the results cannot be entirely ruled out.

## Conclusion

In this cross-sectional study, we uncovered a non-linear relationship of dietary riboflavin intake with depression and suicidal ideation among general American adults. Further analysis revealed that this non-linear relationship was present only in women. The findings of this study might potentially enhance the understanding of micronutrient interventions in patients with depression, thereby providing a basis for the development of more effective health promotion and disease prevention strategies. In the future, the implementation of randomized controlled trials will be essential to validate the reliability of these findings.

## Data Availability

The datasets presented in this study can be found in online repositories. The names of the repository/repositories and accession number(s) can be found in the article/supplementary material.
